# Genetic polymorphisms of *PIP5K2A* and course of schizophrenia

**DOI:** 10.1186/s12881-020-01107-w

**Published:** 2020-10-22

**Authors:** Evgeniya G. Poltavskaya, Olga Yu. Fedorenko, Natalya M. Vyalova, Elena G. Kornetova, Nikolay A. Bokhan, Anton J. M. Loonen, Svetlana A. Ivanova

**Affiliations:** 1 Mental Health Research Institute, Tomsk National Research Medical Center of the Russian Academy of Sciences, Aleutskaya str., 4, Tomsk, Russian Federation 634014; 2 National Research Tomsk Polytechnic University, Tomsk, Russian Federation; 3 National Research Tomsk State University, Tomsk, Russian Federation; 4 Siberian State Medical University Hospital, Moscowsky Trakt, 2, Tomsk, Russian Federation; 5 Groningen Research Institute of Pharmacy, PharmacoTherapy, Epidemiology & Economics, University of Groningen, Antonius Deusinglaan 1, 9713 AV Groningen, The Netherlands; 6 GGZ Westelijk Noord-Brabant, Hoofdlaan 8, 4661 AA Halsteren, The Netherlands

**Keywords:** Schizophrenia, *PIPK2A*, Genetic polymorphism, Type of course, Leading symptoms

## Abstract

**Background:**

Schizophrenia is a severe highly heritable mental disorder. The clinical heterogeneity of schizophrenia is expressed in the difference in the leading symptoms and course of the disease. Identifying the genetic variants that affect clinical heterogeneity may ultimately reveal the genetic basis of the features of schizophrenia and suggest novel treatment targets. *PIP5K2A* (Phosphatidylinositol-4-Phosphate 5-Kinase Type II Alpha) has been investigated as a potential susceptibility gene for schizophrenia.

**Methods:**

In this work, we studied the possible association between eleven polymorphic variants of *PIP5K2A* and the clinical features of schizophrenia in a population of 384 white Siberian patients with schizophrenia. Genotyping was carried out on QuantStudio 5 Real-Time PCR System with a TaqMan Validate SNP Genotyping Assay (Applied Biosystems, USA).

**Results:**

*PIP5K2A* rs8341 (χ^2^ = 6.559, *p* = 0.038) and rs946961 (χ^2^ = 5.976, *p* = 0.049) showed significant association with course of schizophrenia (continuous or episodic). The rs8341*CT (OR = 1.63, 95% CI: 1.04–2.54) and rs946961*CC (OR = 5.17, 95% CI: 1.20–22.21) genotypes were associated with a continuous type of course, while the rs8341*TT genotype (OR = 0.53, 95% CI: 0.29–0.97) was associated with an episodic type of course of schizophrenia. Therefore rs8341*TT genotype presumably has protective effect against the more severe continuous course of schizophrenia compared to the episodic one.

**Conclusions:**

Our experimental data confirm that *PIP5K2A* is a genetic factor influencing the type of course of schizophrenia in Siberian population. Disturbances in the phosphatidylinositol pathways may be a possible reason for the transition to a more severe continuous course of schizophrenia.

## Background

Schizophrenia is a severe and persistent mental disorder involving chronic or recurrent psychosis with a population prevalence of nearly 1% [1]. Clinical symptoms of schizophrenia vary among individuals. The results of the major studies on the course of the illness over 20 to 40 years of follow-up are consistent in reporting a chronic, generally persistent course of illness for 50 to 70% of the patients who receive an initial diagnosis of schizophrenia [2–7]. The continuous course of schizophrenia is characterized by a worse prognosis than episodic. In Ciompi’s classic study [8] about half of patients with schizophrenia had an undulating course, with partial or full remissions followed by recurrences, in an unpredictable pattern. About one-third had relatively chronic, unremitting course with poor outcome. In that study a minority of patients had a steady pattern of recovery with good outcome. Studies on discharge indicated that about 20% of patients don’t require re-admission, many years after discharge. Among these studies, the Danish one [9] is particularly interesting because the sample size and the long follow-up. Following the first discharge, 20% of the surviving patients had not been readmitted after 10 years of follow-up [10].

It is long known that schizophrenia has a large genetic component, with heritability between 64 and 81% [11, 12]. It is characterized by a substantial genetic heterogeneity with contributions from common, rare, and de novo variants of a large number of genes, in addition to environmental factors. GWAS results indicate that schizophrenia is a polygenic disorder, for which thousands of common genetic variants with modest individual effects act in aggregate to increase disease liability [13–15].

Recent research has considerably advanced our understanding in terms of identifying over 100 risk loci associated with schizophrenia. However, many questions remain unanswered, including several which affect their individual clinical significance [16].

It is necessary to investigate the genetic architecture of schizophrenia taking into account not only the presence or absence of a diagnosis of schizophrenia but also the duration, type of the clinical course of disease, leading symptoms (positive or negative) with the main goal of identifying reliable predictive markers as well as new therapeutic targets that might improve the life management of patients with schizophrenia.

*PIP5K2A* (Phosphatidylinositol-5-Phosphate 4-Kinase, Type II, Alpha) has been investigated as a potential susceptibility gene for schizophrenia [17–21] and antipsychotic induced tardive dyskinesia [22]. The main product of PIP5K2A, PI(4,5) P_2_, is involved in transmembrane transduction of neurotransmitter signals by regulating functions of numerous neuronal ion channels and transporters [23–25]. PIP5K2A upregulates the KCNQ potassium channels [23], the glutamate transporters EAAT3 [24], and glutamate GluA1 receptor [25] via phosphatidylinositol-4,5-bisphosphate (PIP2) synthesis. KCNQ channels suppress basal activity of dopaminergic neurons and dopaminergic firing. EAAT3 transporters take up excessive glutamate from the extracellular space. GluA1 receptors are some of the most important excitatory receptors in the central nervous system. It has been shown that schizophrenia linked mutation (N251S)-PIP5K2A results in reduced function of KCNQ channels, EAAT3 transporters, GluA1 receptors and thereby might explain the loss of dopaminergic, glutamatergic control in patients with schizophrenia carriers of this mutation [23–25]. In this work, we studied the possible association between eleven polymorphic variants of *PIP5K2A* and the clinical characteristics of schizophrenia in a population of Caucasian Siberian patients with schizophrenia in order to establish the possible role of PIP5K2A in the clinical heterogeneity of schizophrenia.

## Methods

In this study we examined the contribution of *PIP5K2A* polymorphisms to the development of the clinical features of schizophrenia, such as the leading symptoms (negative or positive) and the type of course of schizophrenia. Based upon reviewing the literature we selected a set of eleven polymorphisms in *PIP5K2A* and here we present new data on the association between them and clinical phenotypes in antipsychotic-treated patients with schizophrenia from West Siberia, Russian Federation.

### Patients

The work was carried out in accordance with The Code of Ethics of the World Medical Association (Declaration of Helsinki 1975, revised in Fortaleza, Brazil, 2013) for experiments involving humans. In this study 384 patients from three psychiatric hospitals in Tomsk, Kemerovo, and Chita oblasts in Siberia were retrieved. The inclusion criteria were a clinical diagnosis of schizophrenia according to ICD-10 (F20), patients aged 18–75 years, Caucasian physical appearance and a signed informed consent form to participate in the study after approval from the study (protocol N63/7.2014) from the Local Bioethics Committee of the Mental Health Research Institute. Exclusion criteria for all patients were non-Caucasian physical appearance (e.g., Mongoloid, Buryats or Khakassians), organic mental disorders (e.g., epilepsy, Parkinson’s disease). Clinical examination and diagnostic assessment were carried out using the Positive and Negative Syndrome Scale (PANSS). The total PANSS score in the group of patients with schizophrenia was 102 [92; 109] (Me [Q1; Q3]). The course of schizophrenia (continuous or episodic) was determined by ICD-10 – in the classification of ICD-10, the fifth character is used for this. Demographic and clinical characteristics of patients with schizophrenia are presented in Table 1.

**Table 1:** Demographics and clinical characteristics of patients with schizophrenia

	All (*n* = 384)	Male (*n* = 237)	Female (*n* = 147)	*p*-value*
Age (mean ± SD)	42 (30–53)	39 (28–51)	47 (33–56)	0.000216
Age of beginning of disease	23 (19–31)	23 (19–30)	24 (20–33)	0.116
Duration of disease, years	14.0 (7.0–25.0)	13.0 (6.0–22.0)	15.5 (8.0–30.0)	0.004

*calculated with the Mann-Whitney U test

To study the associations between *PIP5K2A* polymorphisms and leading symptoms (negative or positive) the total group of 384 patients with schizophrenia was divided into 2 subgroups according to the PANSS survey data: a subgroup of 122 patients with leading negative symptoms and a subgroup of 181 patients with leading positive symptoms. The rest of the patients were not included in the comparison due to the mixed symptoms and the lack of prevalence of positive or negative symptoms according to the PANSS. To study the role of *PIP5K2A* polymorphisms in the development of the course of schizophrenia the total group of 384 patients with schizophrenia was divided into 2 subgroups: a subgroup of 269 patients with continuous course of schizophrenia and a subgroup of 115 patients with episodic course of the disease.

Blood samples were taken 8 h after overnight fasting in tubes containing EDTA and stored in several aliquots at -20 °C until DNA isolation.

### DNA analysis

DNA was isolated from the leukocytes in whole peripheral blood from patients with schizophrenia using the standard phenol-chloroform method. During SNPs selection we were guided by two criteria: a) relevance of selected SNPs to associations with schizophrenia and other mental disorders according to the literature data; b) the minor allele frequency (MAF) of selected SNPs should have been 0.05 (5%) or greater. Information on the selected SNPs for *PIP5K2A* is presented in Additional file 1. Genotyping of *PIP5K2A* polymorphisms (rs10828317, rs8341, rs746203, rs10430590, rs946961, rs1132816, rs1417374, rs943190, rs943194, rs1171506, rs11013052) was carried out on QuantStudio 5 Real-Time PCR System with a TaqMan Validate SNP Genotyping Assay (Applied Biosystems, USA). Experimental studies were carried at the core facilities centre of research equipment and experimental biological material “Medical genomics” of Tomsk National Research Medical Center.

### Statistical analysis

Statistical analysis was performed using SPSS software for Windows, release 21. The genotypes were checked for Hardy–Weinberg equilibrium using chi-square test. Chi-square test and the Fisher’s exact test, where necessary, were used for between-group comparisons of genotypes or allele frequencies. Pairwise linkage disequilibrium (LD) was computed in Haploview program, version 4.2 [26]. The parameters for LD computation include the correlation coefficient (r2), haplotype estimation using accelerated EM algorithm similar to the partition/ligation method described in Qin et al., 2002 [27].

## Results

### Association of *PIP5K2A* polymorphisms with leading (positive vs. negative) symptoms of schizophrenia

The frequency and haplotype analysis found no difference between genotypes and alleles of *PIP5K2A* polymorphisms in patients with schizophrenia with negative leading symptoms and those with positive leading symptoms (Additional file 2).

### Association of *PIP5K2A* polymorphisms with type of course (continuous vs. episodic) of schizophrenia

From the list of eleven SNPs studied for *PIP5K2A* rs8341 (χ^2^ = 6.559, *p* = 0.038) and rs946961 (χ^2^ = 5.976, *p* = 0.049) showed significant association with course of schizophrenia (continuous or episodic). The rs8341*CT (OR = 1.63, 95% CI: 1.04–2.54) and rs946961*CC (OR = 5.17, 95% CI: 1.20–22.21) genotypes were associated with a continuous type of course, while the rs8341*TT genotype (OR = 0.53, 95% CI: 0.29–0.97) was associated with an episodic type of course of schizophrenia (Table 2). Therefore rs8341*TT genotype presumably has protective effect against the more severe continuous course of schizophrenia compared to the episodic one.

**Table 2:** Frequency distribution of genotypes and alleles of *PIP5K2A* polymorphisms in patients with schizophrenia with different types of course of the disease

SNP	Genotype Allele	Frequency		Chi-square	*p*-value	OR	Cl 95%
		Group of patients with a continuous course of schizophrenia (*n* = 269)	Group of patients with an episodic course of schizophrenia (*n* = 115)				
rs10828317	CC	0.187	0.175	0.072	0.965	1.08	0.61–1.91
	CT	0.403	0.412			0.96	0.62–1.50
	TT	0.410	0.412			0.99	0.64–1.55
	C	0.388	0.382	0.030	0.870	1.03	0.75–1.41
	T	0.612	0.618			0.97	0.71–1.34
rs8341	CC	0.387	0.426	6.559	**0.038**	0.85	0.54–1.32
	CT	0.502	0.383			1.63	1.04–2.54
	TT	0.112	0.191			0.53	0.29–0.97
	C	0.638	0.617	0.280	0.600	1.09	0.79–1.50
	T	0.362	0.383			0.92	0.67–1.26
rs746203	CC	0.133	0.195	4.756	0.093	0.63	0.35–1.14
	CT	0.494	0.381			1.59	1.01–2.50
	TT	0.373	0.425			0.80	0.51–1.26
	C	0.380	0.385	0.010	0.900	0.98	0.71–1.35
	T	0.620	0.615			1.02	0.74–1.41
rs10430590	AA	0.467	0.437	1.754	0.416	0.76	0.46–1.25
	AT	0.434	0.402			1.14	0.68–1.89
	TT	0.099	0.061			1.70	0.62–4.60
	A	0.684	0.738	1.690	0.190	0.77	0.52–1.14
	T	0.316	0.262			1.30	0.87–1.94
rs946961	CC	0.108	0.023	5.976	**0.049**	5.17	1.20–22.21
	CG	0.478	0.529			0.82	0.50–1.33
	GG	0.414	0.448			0.87	0.53–1.42
	C	0.347	0.287	2.100	0.150	1.32	0.91–1.92
	G	0.653	0.713			0.76	0.52–1.10
rs1132816	AA	0.621	0.685	1.959	0.376	0.75	0.45–1.26
	AG	0.294	0.270			1.13	0.66–1.94
	GG	0.085	0.045			1.97	0.66–5.89
	A	0.768	0.820	2.080	0.150	0.73	0.47–1.12
	G	0.232	0.180			1.38	0.89–2.13
rs1417374	AA	0.129	0.151	3.735	0.154	0.83	0.41–1.67
	AG	0.469	0.349			1.65	0.99–2.75
	GG	0.402	0.500			0.67	0.41–1.10
	A	0.363	0.326	0.780	0.380	1.18	0.82–1.71
	G	0.637	0.674			0.85	0.59–1.22
rs943190	CC	0.131	0.209	5.326	0.070	0.57	0.30–1.08
	CT	0.502	0.372			1.70	1.03–2.81
	TT	0.367	0.419			0.80	0.49–1.32
	C	0.382	0.395	0.090	0.760	0.95	0.66–1.35
	T	0.618	0.605			1.06	0.74–1.51
rs943194	GG	0.118	0.123	0.506	0.776	0.95	0.44–2.06
	GT	0.489	0.444			1.20	0.72–1.99
	TT	0.392	0.432			0.85	0.51–1.42
	G	0.363	0.346	0.160	0.690	1.08	0.74–1.57
	T	0.637	0.654			0.93	0.64–1.35
rs1171506	AA	0.097	0.037	2.938	0.230	2.80	0.82–9.55
	AG	0.413	0.432			0.92	0.56–1.54
	GG	0.490	0.531			0.85	0.51–1.40
	A	0.304	0.253	1.510	0.220	1.29	0.86–1.93
	G	0.696	0.747			0.78	0.52–1.16
rs11013052	AA	0.083	0.048	1.762	0.414	1.82	0.61–5.46
	AC	0.365	0.333			1.15	0.68–1.94
	CC	0.552	0.619			0.76	0.46–1.25
	A	0.266	0.214	1.770	0.180	1.33	0.87–2.02
	C	0.734	0.786			0.75	0.50–1.14

Bold text indicates *p*-value< 0.05

We also did *PIP5K2A* haplotype analysis for these variants in pairs, and a significant difference was observed for several haplotypes (Table 3). After the detected haplotypes were adjusted for multiple comparisons (10,000 permutations), there was no significant difference in the distribution for them.

**Table 3:** Association of *PIP5K2A* haplotypes with course of schizophrenia

SNPs	Haplotype	Frequency	Group 1, Group 2 frequencies	Chi square	P_asym_	P_Perm_
rs736203/rs943190	TT	0.571	0.565, 0.585	0.269	0.6043	0.9971
	CC	0.342	0.332, 0.366	0.836	0.3606	0.9437
	TC	0.046	0.053, 0.031	1.715	0.1904	0.7448
	CT	0.040	0.050, 0.018	4.463	**0.0346**	0.2097
rs10430590/rs1132816	AA	0.570	0.557, 0.608	1.397	0.2372	0.5328
	TA	0.211	0.211, 0.212	0.001	0.9750	1.0
	AG	0.127	0.128, 0.126	0.005	0.9451	0.9998
	TG	0.091	0.104, 0.054	3.990	**0.0458**	0.1130
rs943190/rs1132816	TA	0.497	0.501, 0.487	0.108	0.7430	0.9812
	CA	0.285	0.268, 0.334	2.827	0.0927	0.2276
	TG	0.117	0.117, 0.119	0.008	0.9309	0.9998
	CG	0.101	0.115, 0.061	4.276	**0.0386**	0.0946
rs11013052/rs1132816	CA	0.643	0.632, 0.675	1.075	0.2998	0.6804
	AA	0.139	0.137, 0.145	0.079	0.7791	0.9898
	AG	0.114	0.129, 0.073	4.119	**0.0424**	0.1423
	CG	0.104	0.103, 0.107	0.029	0.8652	0.9973
rs1132816/rs943194	AG	0.606	0.595, 0.637	1.004	0.3163	0.9144
	AC	0.175	0.173, 0.183	0.099	0.7535	0.9998
	GC	0.175	0.175, 0.103	5.158	**0.0231**	0.1419
	GG	0.063	0.058, 0.077	0.786	0.3754	0.9502

P_asym_ - asymptotic *P* value, P_Perm_ - empirical P value for 10,000 permutations. Group 1 – group of patients with continuous course of schizophrenia (*n* = 269), Group 2 – group of patients with episodic course of schizophrenia (*n* = 115). Bold text indicates *p*-value< 0.05

## Discussion

Several independent linkage studies using different family samples have suggested that the chromosome region where *PIP5K2A* locates is schizophrenia susceptible [28–42]. Many neurotransmitter receptors which have been attributed to schizophrenia are connected to the phosphatidylinositol (PI) pathway, and genes involved in the PI pathway are potential candidates for schizophrenia susceptibility. PIP5K2A is an important enzyme in the PI pathway, and is therefore significant for schizophrenia study [19]. *PIP5K2A* has been shown to be associated with schizophrenia [17–21]. However, the effect of *PIP5K2A* polymorphisms on the clinical manifestations of the disease has been little investigated. In this study, the course of schizophrenia was studied and attempted to identify associations of genetic polymorphisms of the *PIP5K2A* with the type of course of schizophrenia and the leading symptoms.

**Fig. 1:**
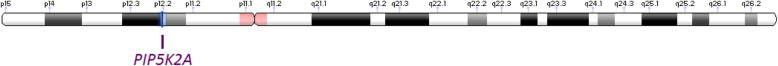
Localization of the *PIP5K2A* gene on chromosome 10

*PIP5K2A* is located on chromosome 10 (Fig. 1). The length of the region comprising the studied polymorphisms is 303 kb.

Only two of the investigated SNPs in this study are located in the exons; they are 10,828,317 in exon 7 and rs1132816 in exon 1. The rest of the studied SNPs are located in intergenic/non-coding regions. However, these polymorphisms are also important. Several association studies have investigated the relationship between genetic variants at *PIP5K2A* and schizophrenia. Sewekow et al. (2003) investigated the linkage region on chromosome 10p12 by analyzing 55 densely spaced genetic variants in 71 schizophrenia families of German origin and found two SNPs rs10828317 and rs1417374 to be significantly associated with schizophrenia [43]. A family-based transmission disequilibrium test involving subjects from the German and Israeli populations found that SNPs rs1417374, rs10828317, rs11013052, rs943190, rs10430590, rs746203 and rs8341 in *PIP5K2A* are significantly associated with schizophrenia [17].

In our work from the list of eleven SNPs studied for *PIP5K2A* none contributed to the development of leading symptoms (positive or negative) of schizophrenia. We obtained data on the association of two SNPs rs8341 and rs946961 with the type of course of schizophrenia (continuous or episodic). The rs8341*CT and rs946961*CC genotypes were associated with a continuous type of course, while the rs8341*TT genotype was associated with an episodic type of course of schizophrenia. Therefore rs8341*TT genotype presumably has protective effect against the more severe continuous course of schizophrenia compared to the episodic one.

**Fig. 2:**
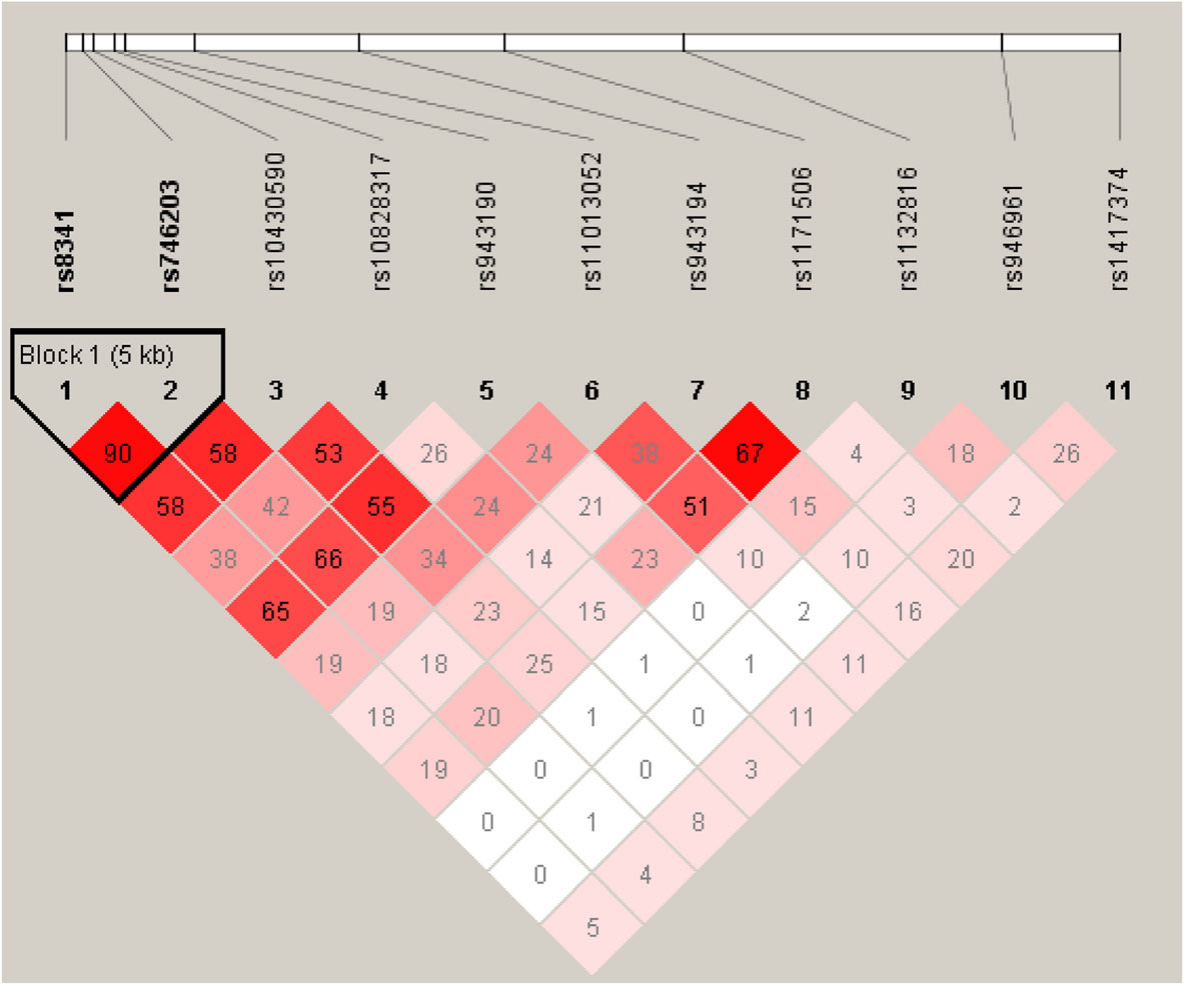
Linkage disequilibrium (LD) pattern for the 11 *PIP5K2A* variants identified in Russian population of patients with schizophrenia. The plot was generated using Haploview 4.2. Pairwise r^2^ values are shown in diamonds that represent the pairwise LD between the 2 SNPs at the top left and right of the corresponding diamond. Colour Scheme: White, shades of pink for D’ < 1; bright red for D’ = 1

Figure 2 represents the Linkage disequilibrium (LD) plot showing the positions of eleven *PIP5K2A* polymorphisms in patients with schizophrenia. LD measures were made with the program Haploview version 4.2.

A linkage disequilibrium pattern was performed for 11 SNPs. The highest value of D’ (0,976) and r^2^ (0.9) was for rs8341 and rs946961 polymorphisms. A haplotype analysis was performed for these SNPs, but we did not reveal any significant associations with the studied clinical characteristics of schizophrenia. In the pairwise analysis of haplotypes, data were obtained on the association of five different haplotypes with the course of schizophrenia. However, after conducting permutation tests, the p level did not reach the significance level. Nevertheless, the data obtained leave open the question about the significance of the polymorphisms included in these haplotypes in the development of the clinical picture of the disease. It is interesting that a synonymous mutation rs1132816 (triplet TST, encoded by serine, is replaced by TCC) is among four of these five haplotypes. Currently, it is believed that synonymous mutations do not affect the function of the protein, since they do not change the amino acid that is encoded by the modified part of the gene. But there is a possibility that such a mutation matters at the stages of transcription or translation of a protein, in any way changing the rate of percentages or the frequency of errors. Nevertheless, it is clear from the data obtained that the rs1132816 polymorphism does not have sufficient power for an independent effect on the course of schizophrenia. Thus, we received confirmation of participation only rs8341 and rs946961 in the development of the schizophrenia clinical phenotypes. PIP5K2A controls the function of neuronal KCNQ potassium channels via phosphatidylinositol-4,5-bisphosphate (PIP2) synthesis [23], which suppress basal activity of dopaminergic neurons and dopaminergic firing. Therefore PIP5K2A modulation of KCNQ potassium channels might influence well dopaminergic neurotransmission in schizophrenia [44]. This regulation could be disrupted in mutant forms of *PIP5K2A*, which may contribute to the schizophrenia phenotype. Moreover, PIP5K2A is a signaling element in the glutamatergic system regulation, specifically it upregulates glutamate transporter EAAT3 [24], which takes up glutamate from the extracellular space, and glutamate GluA1 receptor [25]. It was shown that functionally impaired kinase like PIP5K2A(N251S) may disturb local PIP2 compositions leading to down regulation of EAAT3 [24], and GluA1 [25] and thus be partially effective through deranged glutamate metabolism in the brain of schizophrenic patients carrying this mutation. We assume that this could at least in theory be true for the studied SNPs. Disturbances of the PI path may be a possible reason for the transition to a more severe continuous course of schizophrenia. However, clarification of its possible role in the etiology of schizophrenia will require further studies.

## Conclusions

In conclusion, we found an association of type of course (continuous or episodic) of schizophrenia with *PIP5K2A* rs8341 and rs946961 that confirms *PIP5K2A* to be a genetic factor influencing the type of course of schizophrenia in Siberian population. Strength of our study is the relatively large patient population. A relative weakness is understudying of other risk variants of *PIP5K2A*. Disturbances in the phosphatidylinositol pathways may be a possible reason for the transition to a more severe continuous course of schizophrenia. A further search for genetic markers associated with the development of clinical phenotypes in schizophrenia is needed.

## Supplementary information

**Additional file 1. **Information on the selected SNPs for *PIP5K2A*.

**Additional file 2.** PIP5K2A SNPs in positive vs. negative schizophrenia symptoms.

## Data Availability

The data generated in current study is available in the public repository by identifier 10.6084/m9.figshare.12525401.

## Abbreviations

BDNF: brain derived neurotrophic factor
CI: Lower and upper bound 95% confidence intervals
CNP: 2′3’-cyclonucleotide 3′-phosphodiesterase
HWE: Hardy-Weinberg equilibrium
ICD-10: International Statistical Classification of Diseases and Related Health Problems 10th Revision
LD: Linkage disequilibrium
OR: Odds ratio
PI: phosphatidylinositol
PIP5K2A: Phosphatidylinositol-4-Phosphate 5-Kinase Type II Alpha
SNP: Single nucleotide polymorphism
