# Supplementary material for: Genetic polymorphisms of PIP5K2A and course of schizophrenia
Source: BMC Med Genet. 2020 Oct 22;21(Suppl 1):171. doi: 10.1186/s12881-020-01107-w (PMC7579868; doi:10.1186/s12881-020-01107-w)
Supplement: Supplementary file 1 — Additional file 1. Information on the selected SNPs for PIP5K2A. [file 12881_2020_1107_MOESM1_ESM.docx]

**Additional file 1**

Information on the selected SNPs for *PIP5K2A* is presented in the table S1. In our study, the prevalence of genotypes of all SNPs studied for *PIP5K2A,* except rs10828317 and rs1132816, in group of patients with schizophrenia was consistent with Hardy-Weinberg equilibrium (p≥0.05) (Table S1). It is worth noting that rs10828317 is a functional mutation that changes the protein conformation, which could be a factor significant for natural selection, affecting consistency with Hardy-Weinberg equilibrium. Our results are in agreement with data from the 1000 Genomes database, where the distribution of the rs10828317 genotypes for the entire sample, including the European sample, is not consistent with Hardy-Weinberg equilibrium and a lack of heterozygotes is observed. As for the polymorphic variant rs1132816, it is a synonymous replacement. In the 1000 Genomes database, the distribution of rs1132816 genotypes in the entire sample is also not consistent with the Hardy-Weinberg equilibrium, but the equilibrium is observed for a smaller European sample. Our results may suggest the significance of this polymorphic variant for natural selection, that is line with our data from pairwise haplotype analysis, where this polymorphism occurs in four of the five significant pairs of haplotypes.

**Table S1**

List of analyzed polymorphic variants of *PIP5K2A*

| SNP | Position | Alleles | Minor Allele Frequency | HWE | |
| --- | --- | --- | --- | --- | --- |
|  |  |  |  | Chi-square | p-value |
| rs10828317 | 22550699 | T/C | 24.8 | 7.93 | 0.005 |
| rs8341 | 22536204 | C/T | 26.5 | 0.01 | 0.98 |
| rs746203 | 22541612 | T/C | 46.0 | 0.24 | 0.63 |
| rs10430590 | 22544134 | A/T | 23.4 | 0.03 | 0.87 |
| rs946961 | 22805899 | G/C | 43.0 | 3.88 | 0.05 |
| rs1132816 | 22714297 | A/G | 33.1 | 8.21 | 0.004 |
| rs1417374 | 22839546 | G/A | 23.3 | 0.60 | 0.44 |
| rs943190 | 22553439 | T/C | 34.9 | 0.04 | 0.84 |
| rs943194 | 22620913 | T/G | 46.0 | 0.49 | 0.48 |
| rs1171506 | 22662938 | G/A | 22.6 | 0.05 | 0.83 |
| rs11013052 | 22573558 | C/A | 23.6 | 1.02 | 0.31 |
