# Supplementary material for: Genetic polymorphisms of PIP5K2A and course of schizophrenia
Source: BMC Med Genet. 2020 Oct 22;21(Suppl 1):171. doi: 10.1186/s12881-020-01107-w (PMC7579868; doi:10.1186/s12881-020-01107-w)
Supplement: Supplementary file 2 — Additional file 2. PIP5K2A SNPs in positive vs. negative schizophrenia symptoms. [file 12881_2020_1107_MOESM2_ESM.docx]

**Additional file 2**

**Table S2**

Frequency distribution of genotypes and alleles of *PIP5K2A* polymorphisms patients with schizophrenia with negative or positive leading symptoms

| SNP | Genotype  Allele | Frequency | | Chi-square | p-value | OR | Cl 95% |
| --- | --- | --- | --- | --- | --- | --- | --- |
|  |  | leading negative symptoms (n=122) | leading positive symptoms (n=181) |  |  |  |  |
|  |  |  |  |  |  |  |  |
| rs10828317 | CC CT TT | 0.195  0.441  0.364 | 0.251  0.391  0.358 | 1.426 | 0.490 | 0.72  1.23  1.03 | 0.41 – 1.27  0.77 – 1.97  0.64 – 1.67 |
|  | C  T | 0.415  0.585 | 0.447  0.553 | 0.580 | 0.450 | 0.88  1.14 | 0.63 – 1.23  0.82 – 1.59 |
| rs8341 | CC CT TT | 0.385  0.516  0.098 | 0.365  0.470  0.166 | 2.797 | 0.247 | 1.09  1.21  0.55 | 0.68 – 1.75  0.76 – 1.91  0.27 – 1.12 |
|  | C  T | 0.643  0.357 | 0.599  0.401 | 1.190 | 0.270 | 1.21  0.83 | 0.86 – 1.69  0.59 – 1.16 |
| rs746203 | CC CT TT | 0.117  0.492  0.392 | 0.192  0.452  0.356 | 3.005 | 0.223 | 0.56  1.17  1.17 | 0.28 – 1.09  0.74 – 1.87  0.72 – 1.88 |
|  | C  T | 0.363  0.638 | 0.418  0.582 | 1.850 | 0.170 | 0.79  1.26 | 0.56 – 1.11  0.90 – 1.77 |
| rs10430590 | AA AT TT | 0.485  0.465  0.050 | 0.447  0.440  0.113 | 3.048 | 0.218 | 1.17  1.11  0.41 | 0.70 – 1.95  0.66 – 1.85  0.14 – 1.15 |
|  | A  T | 0.718  0.282 | 0.667  0.333 | 1.430 | 0.230 | 1.27  0.79 | 0.86 – 1.89  0.53 – 1.17 |
| rs946961 | CC CG  GG | 0.095  0.552  0.352 | 0.074  0.510  0.416 | 1.192 | 0.551 | 1.32  1.19  0.76 | 0.54 – 3.23  0.72 – 1.96  0.46 – 1.28 |
|  | C  G | 0.371  0.629 | 0.329  0.671 | 0.990 | 0.320 | 1.21  0.83 | 0.83 – 1.75  0.57 – 1.20 |
| rs1132816 | AA AG GG | 0.598  0.324  0.078 | 0.649  0.291  0.061 | 0.738 | 0.691 | 0.81  1.17  1.31 | 0.48 – 1.36  0.68 – 2.02  0.49 – 3.53 |
|  | A  G | 0.760  0.240 | 0.794  0.206 | 0.820 | 0.370 | 0.82  1.22 | 0.54 – 1.26  0.79 – 1.87 |
| rs1417374 | AA AG GG | 0.110  0.460  0.430 | 0.116  0.476  0.408 | 0.118 | 0.943 | 0.95  0.94  1.09 | 0.42 – 2.11  0.56 – 1.56  0.65 – 1.83 |
|  | A  G | 0.340  0.660 | 0.354  0.646 | 0.100 | 0.750 | 0.94  1.06 | 0.65 – 1.37  0.73 – 1.55 |
| rs943190 | CC CT TT | 0.125  0.500  0.375 | 0.182  0.473  0.345 | 1.520 | 0.468 | 0.64  1.11  1.14 | 0.31 – 1.31  0.67 – 1.84  0.68 – 1.92 |
|  | C  T | 0.375  0.625 | 0.419  0.581 | 0.980 | 0.320 | 0.83  1.20 | 0.58 – 1.20  0.84 – 1.73 |
| rs943194 | GG  GT  TT | 0.122  0.490  0.388 | 0.095  0.496  0.409 | 0.476 | 0.788 | 1.33  0.97  0.92 | 0.58 – 3.06  0.58 – 1.64  0.54 – 1.56 |
|  | G  T | 0.367  0.633 | 0.343  0.657 | 0.290 | 0.590 | 1.11  0.90 | 0.76 – 1.63  0.61 – 1.32 |
| rs1171506 | AA AG GG | 0.069  0.392  0.539 | 0.088  0.415  0.497 | 0.584 | 0.747 | 0.76  0.91  1.19 | 0.29 – 1.98  0.54 – 1.52  0.72 – 1.97 |
|  | A  G | 0.265  0.735 | 0.296  0.704 | 0.580 | 0.450 | 0.86  1.17 | 0.57 – 1.28  0.78 – 1.74 |
| rs11013052 | AA  AC  CC | 0.067  0.333  0.600 | 0.089  0.342  0.568 | 0.503 | 0.778 | 0.73  0.96  1.14 | 0.28 – 1.90  0.56 – 1.63  0.68 – 1.90 |
|  | A  C | 0.233  0.767 | 0.260  0.740 | 0.470 | 0.490 | 0.86  1.16 | 0.57 – 1.31  0.76 – 1.75 |
